# Pre-operative Brain Imaging Using Functional Near-Infrared Spectroscopy Helps Predict Cochlear Implant Outcome in Deaf Adults

**DOI:** 10.1007/s10162-019-00729-z

**Published:** 2019-07-08

**Authors:** Carly A. Anderson, Ian M. Wiggins, Pádraig T. Kitterick, Douglas E. H. Hartley

**Affiliations:** 1 National Institute for Health Research (NIHR), Nottingham Biomedical Research Centre, Ropewalk House, 113 The Ropewalk, Nottingham, NG1 5DU UK; 2 Hearing Sciences, Division of Clinical Neuroscience, School of Medicine, University of Nottingham, Nottingham, NG7 2UH UK; 3 Nottingham University Hospitals NHS Trust, Derby Road, Nottingham, NG7 2UH UK

**Keywords:** cochlear implantation, cross-modal plasticity, functional near-infrared spectroscopy, prognostic imaging, speechreading, superior temporal cortex

## Abstract

Currently, it is not possible to accurately predict how well a deaf individual will be able to understand speech when hearing is (re)introduced via a cochlear implant. Differences in brain organisation following deafness are thought to contribute to variability in speech understanding with a cochlear implant and may offer unique insights that could help to more reliably predict outcomes. An emerging optical neuroimaging technique, functional near-infrared spectroscopy (fNIRS), was used to determine whether a pre-operative measure of brain activation could explain variability in cochlear implant (CI) outcomes and offer additional prognostic value above that provided by known clinical characteristics. Cross-modal activation to visual speech was measured in bilateral superior temporal cortex of pre- and post-lingually deaf adults before cochlear implantation. Behavioural measures of auditory speech understanding were obtained in the same individuals following 6 months of cochlear implant use. The results showed that stronger pre-operative cross-modal activation of auditory brain regions by visual speech was predictive of poorer auditory speech understanding after implantation. Further investigation suggested that this relationship may have been driven primarily by the inclusion of, and group differences between, pre- and post-lingually deaf individuals. Nonetheless, pre-operative cortical imaging provided additional prognostic value above that of influential clinical characteristics, including the age-at-onset and duration of auditory deprivation, suggesting that objectively assessing the physiological status of the brain using fNIRS imaging pre-operatively may support more accurate prediction of individual CI outcomes. Whilst activation of auditory brain regions by visual speech prior to implantation was related to the CI user’s clinical history of deafness, activation to visual speech did not relate to the future ability of these brain regions to respond to auditory speech stimulation with a CI. Greater pre-operative activation of left superior temporal cortex by visual speech was associated with enhanced speechreading abilities, suggesting that visual speech processing may help to maintain left temporal lobe specialisation for language processing during periods of profound deafness.

## Introduction

A cochlear implant (CI) can partially restore hearing to profoundly deaf individuals. Whilst cochlear implantation improves speech understanding for most users, large individual variability in CI outcome exists (Blamey et al. 2013; Lazard et al. 2010; Summerfield and Marshall 1995; UK 2004). Prior to cochlear implantation, estimates of prognosis are used to set and counsel patients’ expectations about their likely clinical outcomes and to inform their decision of whether or not to undergo cochlear implantation. The prognostic information available can also be used to help anticipate and tailor how rehabilitation resources can be optimally allocated and applied to patients. Thus, the ability to accurately predict clinical outcome is of great importance for both CI candidates and their clinical team.

Currently, estimates of CI outcome in adults are based on pre-operative factors that include duration of deafness (Blamey et al. 2013; Holden et al. 2013; Summerfield and Marshall 1995), age-at-onset of deafness (Blamey et al. 2013; Teoh et al. 2004), residual hearing (Gantz et al. 1993; Lazard et al. 2012a) and hearing-aid use (Lazard et al. 2012a), amongst others. However, estimates suggest that these established factors, when taken in combination, can only account for up to 20 % of the variability observed in CI outcome (Lazard et al. 2012a). Therefore, currently, there is no accurate predictor of how an individual will fare with a CI, and identification of an accurate prognostic marker is crucial to help clinicians better predict clinical outcomes.

Differences in brain organisation and how it adapts to auditory deprivation may contribute to cochlear implant outcome. Evidence shows that the brain has a remarkable ability to adapt to sensory deprivation; in profoundly deaf individuals, responses to somatosensory (Auer et al. 2007) and visual stimuli (Dewey and Hartley 2015; Finney et al. 2001) have been observed in auditory brain regions. In deaf white cats, it has been shown that this cross-modal plasticity within auditory brain regions can compensate for deafness by supporting enhanced visual abilities, such as visual localisation and motion detection (Lomber et al. 2010). Likewise, in humans, profoundly deaf individuals can display superior visual speechreading skills compared to normal-hearing listeners (Auer and Bernstein 2007; Rouger et al. 2007) that have been associated with enhanced activation of bilateral superior temporal cortex (STC) by visual speech (Capek et al. 2008) and faster neural processing of visual speech information within the STC (Suh et al. 2009). Whilst this cortical plasticity may prove beneficial for communication following deafness (i.e., by supporting better speechreading), it has also been suggested that these adaptations to deafness may have a detrimental effect on auditory rehabilitation with a CI (Sandmann et al. 2012).

The idea that cortical plasticity could be detrimental to hearing restoration is supported by evidence from visual evoked potential (VEP) studies in experienced adult CI users. These studies found that increased cross-modal activation of the right auditory cortex by non-linguistic visual stimuli was related to poor auditory speech understanding in pre- (Buckley and Tobey 2011) and post-lingually deaf CI users (Sandmann et al. 2012). Furthermore, right superior-temporal PET activation by speechreading, soon after cochlear implantation, was negatively correlated with auditory speech understanding following 6 months of CI use (Strelnikov et al. 2013). However, whether cross-modal activation of auditory cortex by visual speech before implantation is linked with auditory speech understanding with a CI remains unexamined (Anderson et al. 2017a; Campbell et al. 2014; Lyness et al. 2013).

To address this, we used functional near-infrared spectroscopy (fNIRS), an optically based neuroimaging technique. fNIRS uses near-infrared light to non-invasively image the haemodynamic response to neuronal activity (Boas et al. 2014; Huppert et al. 2009). Due to its optical nature, one of the major advantages of fNIRS is its compatibility with the magnetic and electronic components of CIs, making it an ideal imaging modality for testing CI populations, affording long-term and repeated neuroimaging of CI recipients using the same tool both pre- and post-operatively (Anderson et al. 2017a, b). Here, we use fNIRS pre-operatively to investigate the relationship between cortical activation and future CI outcome. Along with the potential for post-operative follow-up of patients, the benefits of using fNIRS pre-operatively in this way include its portability and flexibility that enable patients to be scanned in more comfortable and less constrained environments, as well as its low running costs and short imaging times. All of these factors place fNIRS as a technique that could be readily integrated into clinical practice and CI candidacy assessments, if research shows it to offer valuable prognostic information.

We used fNIRS to measure activation to visual speech within the STC of deaf individuals before cochlear implantation. Firstly, we aimed to understand whether fNIRS measures of cross-modal activation obtained pre-operatively could predict future clinical outcomes for CI candidates. To do so, we examined the relationship between pre-operative cross-modal activation to visual speech and postoperative measurements of auditory speech understanding. Based on available evidence, we hypothesised that greater pre-operative levels of cross-modal activation to visual speech within auditory cortex would predict poorer future speech understanding with a CI. Next, we investigated the influence of pre-operative clinical factors, such as the duration and age at onset of deafness, that are known to influence CI outcome: we examined whether pre-operative brain imaging using fNIRS could offer incremental prognostic information and value above that already provided by these known clinical factors. Lastly, to explore underlying mechanisms of the relationship between pre-operative brain activation and post-operative outcomes, we examined whether greater cross-modal activation to visual speech before implantation was associated with greater speechreading proficiency and weaker cortical response to auditory speech after implantation.

## Materials and Methods

### Participants

The study was approved by the Nottingham one Research Ethics Committee (REC reference: 12/EM/0016) and was sponsored by Nottingham University Hospitals NHS Trust (Research & Innovation reference: 11IH007). All participants were native English speakers with self-reported normal or corrected-to-normal vision, without any known language, cognitive or motor disorder or previous brain injury. Three patients and two control subjects were left handed. All participants gave written informed consent before taking part.

Seventeen adults with bilateral profound deafness who had consented to cochlear implantation were recruited through the Nottingham Auditory Implant Programme. All participants met UK national guidelines for cochlear implantation (NICE 2009). Namely, participants had unaided pure-tone air conduction thresholds of ≥ 90 dB hearing level at 2 and 4 kHz in both ears, a best-aided auditory word recognition score of ≤ 50 % on the Bamford-Kowal-Bench (BKB) sentence test (Bench et al. 1979), and had been deemed suitable CI candidates by the Nottingham Auditory Implant Programme. For clinical characteristics of the sample, see Table 1. All participants were implanted unilaterally with a Cochlear™ Nucleus® 6 device with CP910 sound processor that employed the advanced combination encoder (ACE™) stimulation strategy. None of the participants experienced any complications during their CI surgery and no abnormalities were identified on post-operative X-ray. Furthermore, for all participants, all implantable electrodes were situated within the cochlea and post-operative impedances were within normal range on all electrodes. All participants were stimulated in monopolar configuration, and comfort and threshold levels were estimated for each electrode position by the clinical team according to standard clinical protocols.

**Table 1:** Clinical characteristics of the sample

Subject ID	Age	Onset	Duration	Hearing aid T0	Hearing aid T1	CI Side	CI T1	CI outcome
CI_01	52	51	10 months	Left	Yes	Right	6.1	97
CI_02	37	Birth	37	Bilateral	Yes	Right	7.1	61
CI_03	67	44	23	None	No	Right	6.2	91
CI_04^a^	64	24	40	Bilateral	Yes	Left	6.1	92
CI_05	59	20	39	Left	No	Right	6.4	97
CI_06	38	Birth	38	Bilateral	Yes	Right	6.4	10
CI_07	50	25	25	Bilateral	Yes	Right	5.3	99
CI_08	60	52	8	Bilateral	Yes	Left	6.0	100
CI_09	78	45	33	Bilateral	No	Right	5.7	93
CI_10	70	30	40	Left	No	Left	6.1	64
CI_11	57	3	54	Right	No	Right	6.0	85
CI_12	64	5	59	Bilateral	Yes	Left	6.0	28
CI_13	36	4	32	None	No	Right	6.5	1
CI_14^b^	76	65	11	Right	–	Left	–	–
CI_15	43	42	4 months	Left	No	Left	6.1	88
CI_16	78	43	35	Bilateral	No	Left	6.1	67
CI_17	53	25	28	Bilateral	Yes	Right	6.0	95
Mean (SD) N = 15	56.6 (13.9)						6.1 (0.4)	

Table summarising key clinical characteristics of the CI patients in the study. Age = age at implantation (years); Onset = age at onset of bilateral hearing loss (years); Duration = duration of bilateral hearing loss (years, unless otherwise specified); Hearing aid T0 = side of hearing aid worn during testing at T0; Hearing aid T1 = contralateral hearing aid worn during testing at T1; CI side = side of cochlear implantation; CI T1 = duration of CI use at T1 since activation of CI device (months); CI outcome = auditory speech understanding (% correct) at T1. Original source: Anderson et al. (2017b)

^a^Excluded from neuroimaging analysis due to poor fNIRS data quality

^b^Withdrawn at T1

Seventeen normal-hearing (NH) adults were also recruited to serve as a control group. The group’s mean age (57 years, *SD* = 16.8) was approximately matched to that of the CI users mean age (58 years, *SD* = 13.9). All participants had normal hearing thresholds, defined here as average pure-tone air conduction hearing thresholds of ≤ 20 dB (dB) across frequencies 0.5, 1, 2 and 4 kHz in both ears.

### Experimental Design

Pre-operative brain imaging using fNIRS was conducted at the participants’ earliest convenience after having consented to receive a CI, but before undergoing surgery (T0). At T0, CI users were tested in their best-aided condition, i.e. wearing their hearing aids if they used them in everyday life (see Table 1). Brain imaging was also conducted with NH control subjects to enable group comparisons of cortical activation. Behavioural measures of visual speechreading ability were also obtained at T0 for both groups. Post-operative behavioural measures of auditory speech understanding (CI outcome) were obtained in the same individuals approximately 6 months after activation of their CI device (T1, average duration of CI use = 6.13 months, *SD =* 0.4). At T1, CI users were tested in their best-aided condition wearing their preferred listening devices (i.e. CI and optional contralateral hearing aid). The mean retest interval between T0 and T1 for CI users was 8.2 months (*SD* = 1.2).

### Testing Conditions

Testing was carried out in a double-walled sound-attenuated booth. Participants were seated in front of a visual display unit at a viewing distance of 1 m, with a centrally located Genelec 8030A loudspeaker mounted immediately above and behind the visual display unit. All stimuli were presented using the MATLAB® computing environment (Release 2014b, The MathWorks, Natick, MA). Visual components of the stimuli were presented on the visual display unit. To reflect the typical level of conversational speech, auditory components were presented through the loudspeaker at 65 dB SPL (A-weighted root-mean-square sound pressure level averaged over the duration of each sentence.). This was measured at the listening position with the participant absent using a Brüel & Kjær 2250 sound level metre and free-field microphone (Type 4189). Prior to the commencement of each test, participants were provided with written instructions to ensure understanding and consistency of instructions given.

### fNIRS Data Acquisition

At T0, cortical activation was measured using a continuous-wave fNIRS system (ETG-4000, Hitachi Medical Co., Japan). The ETG-4000 is a commercial system that emits a continuous beam of light into the cortex and samples at a rate of 10 Hz. The system measures simultaneously at two wavelengths, 695 nm and 830 nm, to allow for the separate measurement of changes in oxygenated haemoglobin (HbO) and deoxygenated haemoglobin (HbR) concentrations. This specific choice of wavelengths has been shown to minimise cross-talk error between the two chromophores (Sato et al. 2004). A dense sound-absorbing screen was placed between the fNIRS equipment and the participant to attenuate the fan noise generated by the equipment. This resulted in a steady ambient noise level of 38 dB SPL (A-weighted).

### fNIRS Stimuli

The Institute of Hearing Research (IHR) Number Sentences (Hall et al. 2005) were presented as speech stimuli during the acquisition of fNIRS measurements. The corpus comprised digital audio-visual recordings of 90 sentences, each spoken by both a male and female talker. Each of the sentences contained between four and seven words, three of which were designated keywords. For the purpose of this experiment, the speech material was presented in a visual-only condition (V-ONLY, i.e. speechreading) where the visual component of the recording was shown but the auditory component was muted. The speech material was also presented in an auditory (A-ONLY) and audio-visual (AV) condition that is reported and analysed elsewhere. Rest periods consisted of a uniform background with a fixation cross-presented in place of the talker’s mouth.

### fNIRS Paradigm

Thirty IHR number sentences were randomly selected without replacement for presentation in each of the conditions, with the restriction that an equal number were spoken by the male and female talker in each condition. The speech stimuli were presented in a block design paradigm interleaved with rest periods. Each block comprised six concatenated sentences, evenly spaced to fill a 24-s block duration. Five blocks were presented for each stimulus condition. During these blocks, the participants were instructed to attend to the talker and to always try to understand what the talker was saying. To encourage sustained attention throughout the experiment, an attentional trial was presented after two of the 15 stimulus blocks. These blocks were chosen at random, and therefore, the attentional trials occurred at unpredictable positions within the experimental run. Two seconds after the cessation of a chosen block, two alternative words were presented on either side of the fixation cross; in a two-alternative forced choice task, participants were asked to press one of two buttons to indicate which word had been spoken in the immediately preceding sentence. Following the participant’s response, an additional 5-s rest was added to the start of the ensuing rest period. Rest periods were included to allow the haemodynamic response elicited by the stimulation block to return to a baseline level. The durations of the rest periods were randomly varied between 20 and 40 s in 5 s increments.

Prior to fNIRS scanning, participants first completed a short familiarisation run to ensure that they understood the experimental procedure. During the familiarisation session, one block of each of the conditions was presented. In order to avoid pre-exposure to the experimental stimuli, the familiarisation blocks comprised speech material (BKB sentences (Bench et al. 1979)) that was different from the material presented during the fNIRS measurements and the subsequent behavioural testing. Following each stimulation block, an example of the attentional control task was also presented.

### Optode Placement

Two 3 × 3 optode arrays were placed bilaterally over the participant’s temporal lobes. Together, these comprised ten emitter and eight detector optodes with a fixed inter-optode distance of 30 mm, providing a penetration depth into the cortex of approximately 15 mm (Strangman et al. 2014). This resulted in a total of 24 measurement channels (12 per hemisphere).

The optode arrays were positioned on the participant’s head so as to ensure good coverage of the STC. Optode positioning was guided by the International 10-20 System (Jasper 1958) to promote consistency across participants and test sessions. Specifically, on each side, the lowermost source optode was placed as close as possible to the pre-auricular point, with the uppermost source optode aligned towards Cz. Consistency of optode positioning across test sessions at the individual level was further ensured by reference to photographs taken during the initial testing session.

**Fig. 1:**
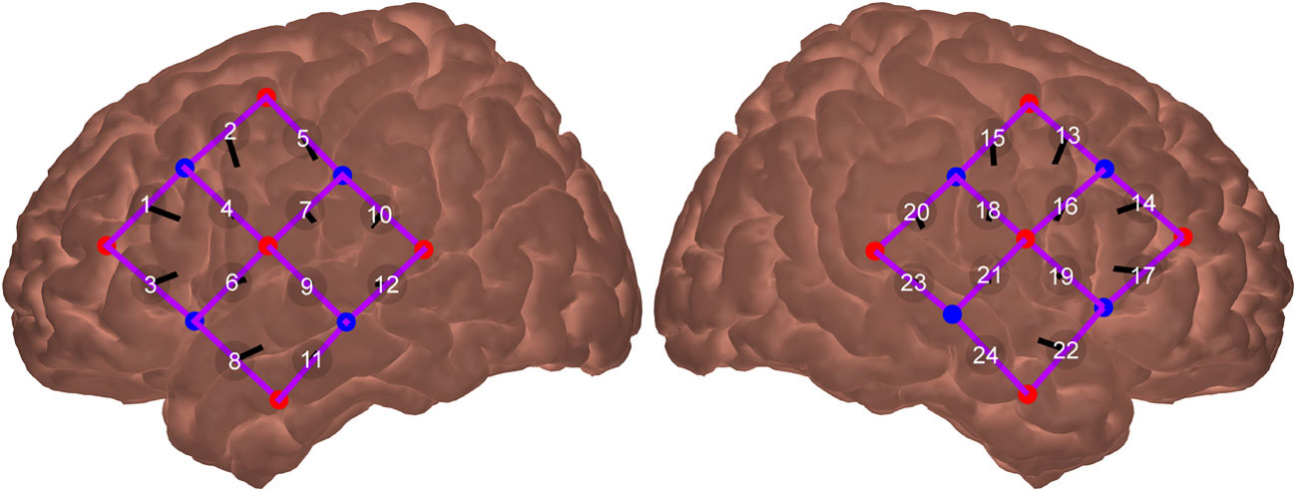
Mean position of fNIRS optodes and measurement channels. Measurement channels are labelled numerically, source optodes are indicated in red and detector optodes are indicated in blue

To evaluate the consistency of optode positioning across individuals, the procedure was piloted on six adult volunteers who did not take part in the main experiment. After positioning the arrays as described above, the optode positions, plus anatomical surface landmarks, were recorded using the Hitachi ETG-4000’s electromagnetic 3D Probe Positioning Unit. For each volunteer, the digitised optode positions were registered to a standard atlas brain, ‘Colin27’(Collins et al. 1998), using the AtlasViewer tool (Aasted et al. 2015), allowing their locations to be visualised relative to underlying cortical anatomy. The standard deviation in the position of each optode was between 2.9 and 8.8 mm. Assessment of the mean optode positions suggested that the array provided good coverage of STC (Fig. 1).

### Definition of Region of Interest

The region of interest (ROI) was the posterior portion of bilateral superior temporal cortex (STC), based on evidence that speech is processed in the temporal lobes bilaterally (Hickok and Poeppel 2007) and that fNIRS responses to speech are also expressed bilaterally in these regions (Wiggins et al. 2016). Examples of deafness-induced cross-modal plasticity have been reported in both hemispheres (Buckley and Tobey 2011; Chen et al. 2016; Doucet et al. 2006; Strelnikov et al. 2013); however, the precise role of plasticity in each hemisphere remains uncertain (Anderson et al. 2017a). Therefore, in the first instance, we examined activation bilaterally. However, recognising that each hemisphere has a different specialisation with regard to speech processing (Cardin et al. 2013; Hall et al. 2005; Lazard et al. 2012b; Zatorre and Belin 2001), in follow-up analyses, we examined each hemisphere separately.

In order to assess the sensitivity of our fNIRS measurements to the underlying cortical regions, using the AtlasViewer tool (Aasted et al. 2015), a Monte Carlo code for simulating the probabilistic path of photon migration through the head (Boas et al. 2002) (‘tMCimg’) was run with 1 × 10^7^ simulated photons launched from each optode position. The resultant sensitivity profiles suggested that channels #9, 10 and 12 (left hemisphere) and channels #20, 21 and 23 (right hemisphere) provided appropriate sensitivity to the posterior portion of STC (as reported in references (Anderson et al. 2017b; Wiggins et al. 2016)).

### Behavioural Test of Speech Understanding

The CUNY (City University of New York) Sentence Lists (Boothroyd et al. 1985) were employed to obtain a measure of speech understanding. The CUNY corpus was employed primarily due to its routine use as a clinical outcome measure by CI programmes across the UK. Additionally, this corpus was not presented during fNIRS scanning, thus helping to limit training effects within and across testing sessions. The CUNY Sentence Lists include 25 standardised lists each comprising 12 sentences that vary in length and topic. Each list contains between 101 and 103 words spoken by a male talker. Two CUNY lists (i.e. 24 sentences) were randomly selected without replacement for presentation in each stimulation condition. Speech understanding was measured in A-ONLY, V-ONLY and AV conditions. However, for the purposes of the present study, we focus only on speechreading ability before implantation (T0) and auditory ability following 6 months of CI use (T1) as a measure of CI outcome. Whilst AV speech recognition is important in everyday life to CI users, traditionally, both pre-operative CI candidacy and post-operative CI outcome are assessed by A-ONLY performance in UK clinics. Separate analysis of AV speech recognition using an additive model is fully reported in CAA’s doctoral thesis (Anderson 2016).

The 24 sentences were presented in random order. After each sentence presentation, the participant was instructed to repeat back all words that they were able to identify. All words correctly reported by the participant were recorded by the researcher on a scoring laptop before initiation of the next trial. The scoring method ignored errors of case or declensions. Prior to commencement of speech understanding testing, all participants completed a short familiarisation run. BKB sentences were employed during the familiarisation run in order to avoid pre-exposure to the CUNY corpus.

### Pre-processing of fNIRS Data

We used analysis methods similar to those used in a number of previous studies conducted in our laboratory (Dewey and Hartley 2015; Wiggins and Hartley 2015; Wiggins et al. 2016). Raw fNIRS recordings were exported from the Hitachi ETG-4000 into MATLAB for use with routines provided in the HOMER2 package (Huppert et al. 2009) and custom scripts. Raw light intensity measurements were first converted to change in optical density (Huppert et al. 2009). Wavelet motion correction was then performed to reduce the impact of motion artefacts on the fNIRS signal. Wavelet filtering can enhance data yield and has emerged as a favourable approach for use with fNIRS data (Molavi and Dumont 2012). The HOMER2 hmrMotionCorrectWavelet function (based on Molavi and Dumont 2012) was used which assumes that the wavelet coefficients have a Gaussian probability distribution and so applies a probability threshold to remove outlying wavelet coefficients that are assumed to correspond to motion artefacts. A probability threshold was set to exclude coefficients lying more than 1.5 inter-quartile ranges below the first quartile or above the third quartile.

Following motion-artefact correction, a bandpass filter of 0.01–0.5 Hz was applied to reduce sources of physiological noise in the data including high-frequency cardiac oscillations, low-frequency respiration and blood pressure changes. The fNIRS signal was next converted into estimates of changes in HbO and HbR using the modified Beer-Lambert law with a default differential path-length factor of six (Huppert et al. 2009). As bandpass filtering is unable to remove all physiological noise from fNIRS recordings (Huppert et al. 2009), the haemodynamic signal separation method of Yamada et al. (Yamada et al. 2012) was also applied. This algorithm separates the fNIRS signal into estimates of the functional and systemic components, based on expected differences in the correlation between HbO and HbR in each component. Specifically, a positive correlation between changes in HbO and HbR is assumed in the systemic component, whereas a negative correlation is assumed in the functional component. The functional component of the signal was identified by the algorithm, extracted from the fNIRS signal and retained for further analysis.

In order to quantify the level of cortical activation, the pre-processed fNIRS signal was subjected to an ordinary least squares general linear model (GLM). The GLM design matrix included three boxcar regressors, one for each stimulation condition. The two response periods following the two attentional trials were also modelled in the design matrix as transient events occurring at the time the two words were presented on screen. All regressors were convolved with the canonical haemodynamic response function provided in SPM8 (http://www.fil.ion.ucl.ac.uk/spm). After completing the first-stage OLS estimation at the single-subject level, we used the Cochrane-Orcutt procedure (Cochrane and Orcutt 1949) to correct for serial correlation. Briefly, this involved fitting a first-order autoregressive process to the model residuals and transforming the original model according to the estimated autoregressive parameter (see Plichta et al. 2007). We then re-estimated the beta weights based on the transformed model (second stage).

The beta weights of the canonical HRF term were extracted for each stimulation condition, at each measurement channel, and for each participant. As described above, the haemodynamic signal separation method employed here (Yamada et al. 2012) assumes a fixed linear relationship between HbO and HbR in the functional response. Therefore, the results of all statistical analyses are identical regardless of whether conducted on the beta weights extracted for the HbO or HbR parameter. For simplicity, only results pertaining to the beta estimates of the HbO parameter of the functional component are presented here. These beta weights were used to quantify the amplitude of cortical activation to speech compared to rest. The resultant beta weights were averaged across the ROI measurement channels and were subjected to further statistical analysis as outlined below.

### Pre-processing of Behavioural Data

Auditory speech understanding and speechreading ability, measured using the CUNY Sentence Lists, were quantified as the percentage of words reported correctly (% correct). In order to make the data more suitable for statistical analysis, the rationalised arcsine transform (Studebaker 1985) was applied using Matlab. Firstly, the arcsine transform (*T*) was applied as follows:

$$ T=\mathrm{arcsine}\sqrt{\frac{X}{N+1}}+\mathrm{arcsine}\sqrt{\frac{X+1}{N+1}} $$

The ‘asin’ function in Matlab was used to return the inverse sine (arcsine) for each value of *X*, where *X* represents the total number of words reported correctly and *N* represents the total number of words presented. This was then transformed linearly:

$$ R=46.47324337T-23 $$

where *R* indicates the resulting rationalised arcsine-transformed score (rationalised arcsine unit, RAU). This transformation extends the original percent correct scale outwards in both directions from 50 %, creating bigger differences as the extremes of the range are approached. Consequently, this transformation makes the rationalised arcsine scale linear and additive in its proportions whilst producing values close to the original percentage scores for values between approximately 15 and 85 % (Studebaker 1985). Subsequently, the transformed scores were subjected to statistical analysis.

### Statistical Analysis

Following the pre-processing of neuroimaging and behavioural data, resultant data were analysed using IBM® SPSS® Statistics software (Release 22.0, Armonk, NY: IBM Corp.). Bivariate linear regression analysis was performed to test whether bilateral STC response to visual speech before implantation was predictive of future CI outcome. Normality of the distribution of bilateral STC activation to visual speech was confirmed. Whilst the Kolmogorov-Smirnov test indicated that the distribution of CI outcome data did not significantly differ from normality, visual inspection of the histogram did indicate slight negative skew, despite applying the rationalised arcsine transform to the raw performance data. This skew was somewhat anticipated given the significant benefits that cochlear implantation can provide, particularly within the first 6 months following implantation (Lenarz et al. 2012). However, post-hoc diagnostic measures of the regression model verified that the assumptions of bivariate linear regression were met: a scatterplot indicated linearity between the predictor and dependent variable, visual inspection of histograms and normal P-P (probability-probability) plots indicated that the standardised residuals of the regression model were normally distributed and that the assumption of homoscedasticity was met.

Multiple regression was conducted to examine whether pre-implant STC activation to visual speech provided incremental predictive value above that of influential clinical characteristics (covariates). For each regression model conducted, the covariate/s of interest was first entered as a predictor variable into block 1, with pre-implant STC activation to visual speech then entered as a predictor into block 2 of the model. For all models, histogram and scatterplots confirmed that the standardised residuals were normally distributed and the assumption of homoscedasticity was met. Furthermore, the Durbin-Watson statistic indicated that the assumption of independent errors was met, and the variance inflation factor indicated that multicollinearity was low between the predictor variables in block 2 of the models and was not problematic.

All data are publicly available through the University of Nottingham’s Research Data Management Repository (10.17639/nott.322).

## Results

### Does Cross-modal Activation to Visual Speech Predict CI Outcome?

As anticipated, a high level of variability in CI outcome was observed across the group of CI users, with auditory performance ranging from 1 to 100 % correct after 6 months of CI use. Both pre-operative brain imaging and postoperative CI outcome data were available for 15 CI users: one participant displayed excessive motion and poor contact between fNIRS optodes and the scalp resulting in poor data quality. This participant was therefore not included in any analysis involving brain imaging data. Another CI user was withdrawn from the study at T1 for unrelated medical reasons and was therefore not included in the outcome prediction analysis.

**Fig. 2:**
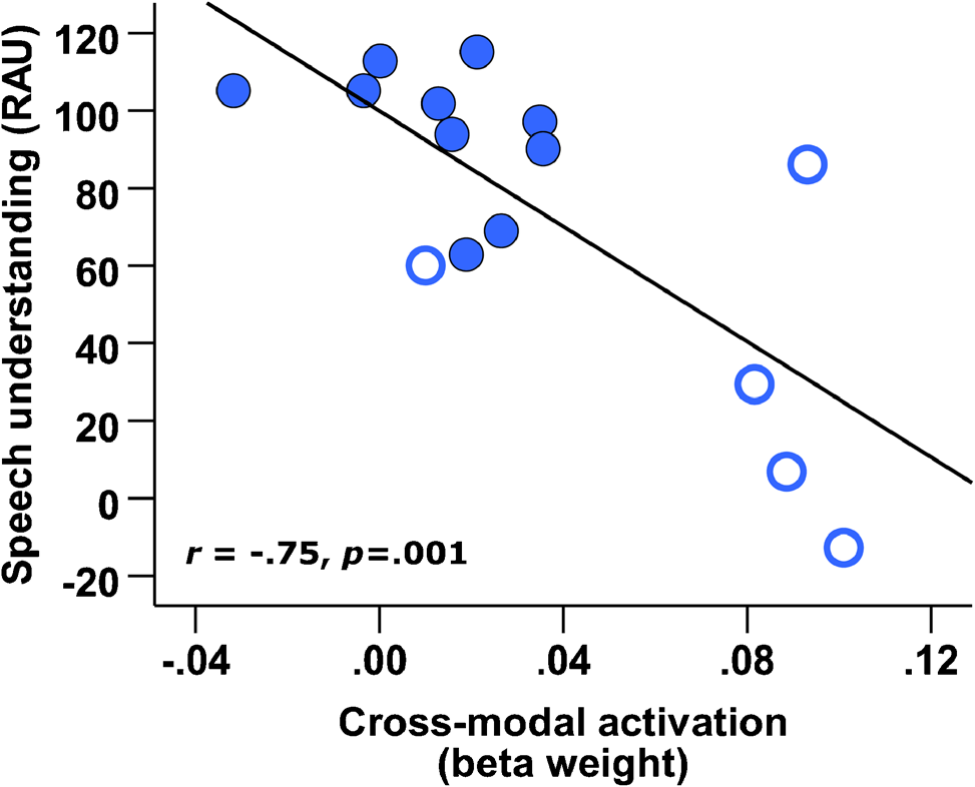
Pre-implant STC activation to visual speech predicts CI outcome. Scatterplot of bilateral STC activation to visual speech before implantation and future CI outcome, with best fitting regression line shown (*n* = 15). Filled markers represent data obtained from post-lingually deaf CI users, and open markers represent data obtained from pre- and peri-lingually deaf CI users

Bivariate linear regression analysis revealed that bilateral STC activation to visual speech before implantation was significantly predictive of future CI outcome, *F*_(1,13)_ = 16.59, *p =* .001 (Table 2, model A). Furthermore, cortical activation to visual speech was able to explain 56 % of the variance observed in CI outcome (*R*^2^ = .56), with an adjusted *R*^2^ of .53, indicating good generalizability of the regression model. In line with our hypothesis, Fig. 2 illustrates that a negative relationship existed (Pearson’s correlation coefficient *r* = − .75, *p* = .001, 2-tailed), whereby individuals showing greater bilateral superior temporal cortex (bSTC) activation to visual speech before implantation had poorer auditory speech understanding following 6 months of CI use. We next conducted separate regression analysis of cortical activation to visual speech within the left and right STC (Table 2, models B and C). This confirmed that the predictive relationship was not driven predominantly by one cerebral hemisphere (left STC *r* = − .68, *F*_(1,13)_ = 10.85, *p =* .006, 2-tailed; right STC *r* = − .55, *F*_(1,13)_ = 5.69, *p* = .033, 2-tailed).

**Table 2:** Summary of bivariate regression statistics for STC activation in the prediction of CI outcome

Dependent *CI OUTCOME*	*R* ^2^	Adj. *R*^2^	*F*	*b*	SE *b*	*β*	*t*
Model A	.56	.53	16.59 (*p* = .001)				
*Constant*				99.88	9.30	–	10.74 (*p* = .000)
*bSTC ACTIVATION*				− 743.47	182.56	− .75	− 4.07 (*p* = .001)
Model B	.46	.41	10.85 (*p* = .006)				
*Constant*				98.49	10.58	–	9.31 (*p* = .000)
*lSTC ACTIVATION*				− 642.91	195.16	− .68	− 3.29 (*p* = .006)
Model C	.30	.25	5.69 (*p* = .033)				
*Constant*				86.78	10.10	–	8.59 (*p* = .000)
*rSTC ACTIVATION*				− 384.50	161.24	− .55	− 2.39 (*p* = .033)

*P* value (2-tailed), *n* = 15. Model A = bilateral STC (bSTC), Model B = left STC (lSTC), and Model C = right STC (rSTC) activation to visual speech before implantation

Here, analysis was conducted across the whole group of CI patients (*n* = 15) as this participant group is representative of the heterogeneous population that present to clinical CI programmes. Whilst we know that one of the most significant predictors of CI outcome is the age at which the onset of deafness occurs, this variable can only account for a small proportion of the overall variance in outcome in pre- and post-lingually deaf individuals (Summerfield and Marshall 1995). Furthermore, when the onset of deafness occurs (pre- or post-lingually), it can influence the extent of cortical plasticity that takes place and the association with future CI outcome (Buckley and Tobey 2011). Indeed, it is apparent from Fig. 2 that group differences between pre- and post-lingually deaf individuals seem to be driving the predictive relationship observed here between cortical activation and CI outcome. To investigate this further, we next removed the five pre-lingually deaf subjects from the analysis. Bivariate linear regression analysis showed that the predictive relationship between activation to visual speech and CI outcome could not be replicated in the remaining subgroup of post-lingually deaf individuals (*n* = 10; bilateral STC *r* = − .41, *F*_(1,8)_ = 1.576, *p =* .245, 2-tailed; left STC *r* = − .02, *F*_(1,8)_ = .005, *p =* .947, 2-tailed; right STC *r* = − .33, *F*_(1,8)_ = .982, *p* = .351, 2-tailed). Therefore, the result appears to be driven by the subgroup of pre-lingually deaf individuals. Subsequently, confounding factors including the duration and age-at-onset of deafness are further explored in following analyses.

### Can Measuring Cortical Activation Provide Additional Prognostic Value?

To investigate whether the pre-operative cortical measure of bilateral STC activation to visual speech could offer incremental prognostic value above that of known clinical factors influencing CI outcome, we next considered its predictive ability when controlling for influential pre-operative characteristics of the CI candidates, including the age-at-onset and duration of deafness prior to cochlear implantation (Blamey et al. 2013; Green et al. 2007; Lazard et al. 2012a; Summerfield and Marshall 1995; Teoh et al. 2004). Indeed, in Fig. 2, it can be seen that those individuals displaying the highest levels of pre-implant STC activation to visual speech and poorer CI outcome were pre- and peri-lingually deafened, whereas individuals displaying the lowest levels of pre-implant STC activation to visual speech and better CI outcome were predominantly post-lingually deafened. Furthermore, we have seen that the predictive relationship between activation to visual speech and CI outcome observed here could not be replicated when examining post-lingually deaf individuals alone. In addition, existing research has also demonstrated positive associations between speechreading ability and the amplitude of temporal lobe response to visual speech in pre-lingually (Capek et al. 2008; Capek et al. 2010) and post-lingually deaf adults (Lee et al. 2007). However, the relationship between pre-implant speechreading ability and CI outcome is unclear, as both positive and negative relationships are reported in the literature (Gantz et al. 1993; Hay-McCutcheon et al. 2005), respectively).

Subsequently, we examined (1) the age-at-onset of bilateral hearing loss, (2) the duration of bilateral hearing loss prior to implantation and (3) the CI candidate’s pre-implant speechreading ability as potential covariates that could have predictive power and influence the relationship between pre-implant cortical activation and future CI outcome. A Pearson’s correlation matrix was used to examine the relationships between these clinical characteristics with (i) pre-implant STC activation to visual speech and (ii) CI outcome (Table 3). This confirmed that associations between the covariates and predictor and dependent variable existed in the anticipated directions.

**Table 3:** Correlations of covariates with cortical activation and CI outcome

		Covariates			Predictor	Dependent
		ONSET	DURATION	SPEECHREADING	bSTC ACTIVATION	CI outcome
Covariates	ONSET	–	− .72 (*p* = .002)	− .56 (*p* = .029)	− .63 (*p* = .013)	.67 (*p* = .007)
	DURATION		–	.60 (*p* = .018)	.55 (*p* = .034)	− .46 (*p* = .086)
	SPEECHREADING			–	.57 (*p* = .026)	− .40 (*p* = .141)
Predictor	bSTC ACTIVATION				–	− .75 (*p* = .001)
Dependent	CI OUTCOME					–

Pearson’s correlation coefficient (*P* value), 2-tailed (not corrected for multiple comparisons), all *n* = 15

ONSET = age at onset of bilateral hearing loss; DURATION = duration of bilateral hearing loss; SPEECHREADING = pre-implant speechreading ability; bSTC ACTIVATION = pre-implant bilateral superior temporal cortex activation to visual speech; CI OUTCOME = auditory speech understanding after 6 months of CI use

Separate hierarchical linear regressions were conducted to estimate the ability of bSTC activation to predict CI outcome independently of each covariate. The regression models indicated that including bSTC activation as a predictor added significant incremental predictive value above that of each of the covariates. Specifically, bSTC activation accounted for an additional 18 % of the total variance in CI outcome above that already explained by the age-at-onset of deafness (Δ*R*^*2*^ = .18, Δ*F*_(1,12)_ = 5.78, *p* = .033; Table 4), an additional 35 % of the variance above that explained by the duration of deafness (Δ*R*^*2*^ = .35, Δ*F*_(1,12)_ = 9.73, *p* = .009; Table 5) and an additional 40 % of the variance above that explained by speechreading ability (Δ*R*^*2*^ = .40, Δ*F*_(1,12)_ = 11.03, *p* = .006; Table 6). Furthermore, the standardised beta coefficients (*β*) of bSTC activation were significant in each regression model, indicating that pre-implant bSTC activation to visual speech was a significant individual predictor of CI outcome when controlling for the effects of the said covariate (see Tables 4, 5 and 6).

**Table 4:** Summary of hierarchical regression statistics when controlling for age-at-onset of bilateral hearing loss

Dependent *CI OUTCOME*		*R* ^2^	Adj. *R*^2^	*F*	Δ*R*^2^	Δ*F*	*b*	SE *b*	*β*	*t*
Model 1	Block 1	.44	.40	10.40 (*p* = .007)	–	–				
	*Constant*						40.24	13.29	–	3.03 (*p* = .010)
	*ONSET*						1.33	.41	.67	3.23 (*p* = .007)
	Block 2	.63	.56	10.00 (*p* = .003)	.18	5.78 (*p* = .033)				
	*Constant*						76.16	18.77	–	4.06 (*p* = .002)
	*ONSET*						.65	.45	.33	1.44 (*p* = .176)
	*bSTC ACTIVATION*						541.12	224.99	.55	− 2.41 (*p* = .033)

*P* value (2-tailed), *n* = 15. ONSET = age at onset of bilateral hearing loss; bSTC ACTIVATION = pre-implant bilateral superior temporal cortex activation to visual speech

**Table 5:** Summary of hierarchical regression statistics when controlling for duration of bilateral hearing loss

Dependent *CI OUCTOME*		*R* ^2^	Adj. *R*^2^	*F*	Δ*R*^2^	Δ*F*	*b*	SE *b*	*β*	*t*
Model 2	Block 1	.21	.15	3.45 (*p* = .086)	–	–				
	*Constant*						106.77	19.56	–	5.46 (*p* = .000)
	*DURATION*						− 1.06	.57	.46	− 1.86 (*p* = .869)
	Block 2	.56	.49	7.75 (*p* = .007)	.35	9.73 (*p* = .009)				
	*Constant*						103.30	15.17	–	6.81 (*p* = .000)
	*DURATION*						− .15	.53	.07	− .29 (*p* = .775)
	*bSTC ACTIVATION*						707.02	226.63	.71	− 3.12 (*p* = .009)

*P* value (2-tailed), *n* = 15. DURATION = duration of bilateral hearing loss; bSTC ACTIVATION = pre-implant bilateral superior temporal cortex activation to visual speech

**Table 6:** Summary of hierarchical regression statistics when controlling for pre-implant speechreading ability

Dependent *CI OUTCOME*		*R* ^2^	Adj. *R*^2^	*F*	Δ*R*^2^	Δ*F*	*b*	SE *b*	*β*	*t*
Model 3	Block 1	.16	.09	2.46 (*p* = .141)	–	–				
	*Constant*						86.48	12.17	–	7.11 (*p* = .000)
	*SPEECHREADING*						− 0.74	0.47	.40	− 1.57 (*p* = .141)
	Block 2	.56	.49	7.70 (*p* = .007)	.40	11.03 (*p* = .006)				
	*Constant*						99.43	9.94	–	10.00 (*p* = .000)
	*SPEECHREADING*						0.08	0.43	.05	0.19 (*p* = .851)
	*bSTC ACTIVATION*						768.87	231.47	.77	− 3.32 (p = .006)

*P* value (2-tailed), *n* = 15. SPEECHREADING = pre-implant speechreading ability; bSTC ACTIVATION = pre-implant bilateral superior temporal cortex activation to visual speech

### Mechanisms Underlying the Predictive Relationship

To investigate the mechanisms underlying the observed predictive relationship between pre-implant cortical activation and future CI outcome, we next explored whether this negative relationship with CI outcome was due to the recruitment of auditory brain regions by visual speech limiting the same regions’ ability to respond to auditory speech stimulation with an implant. Correlational analysis revealed no evidence that greater bSTC activation to visual speech before implantation was associated with smaller bSTC activation to auditory speech 6 months after implantation (*r =* − .03, *p =* .93, 2-tailed, *n* = 15). This suggests that a stronger STC response to visual speech during deafness does not preclude future activation of the same cortical regions by auditory stimulation with a CI.

**Fig. 3:**
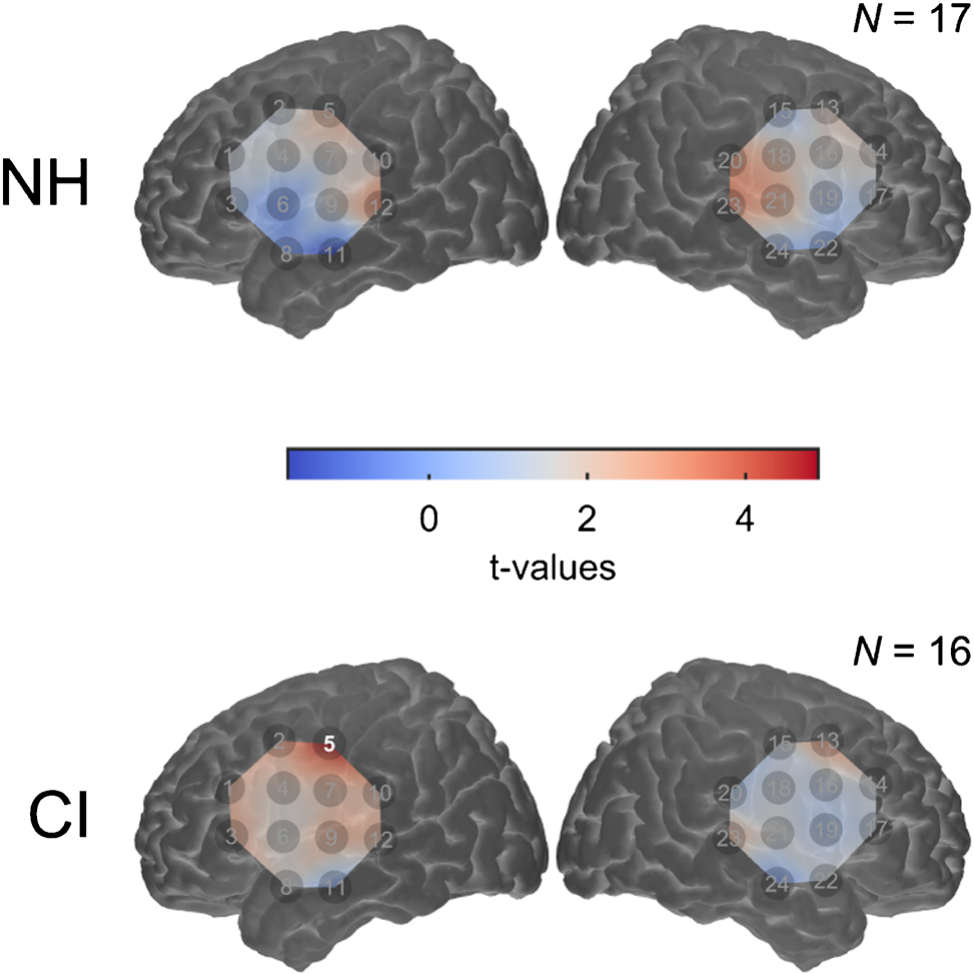
Group-level cortical activation map for visual speech. Amplitude of cortical activation to visual speech for normal-hearing controls (NH, *n* = 17) and CI users before implantation (CI, *n* = 16), colour coded by *t* value. Significantly activated channels releveled by one-tailed *t* tests (*p* < .05, FDR corrected) are highlighted

**Fig. 4:**
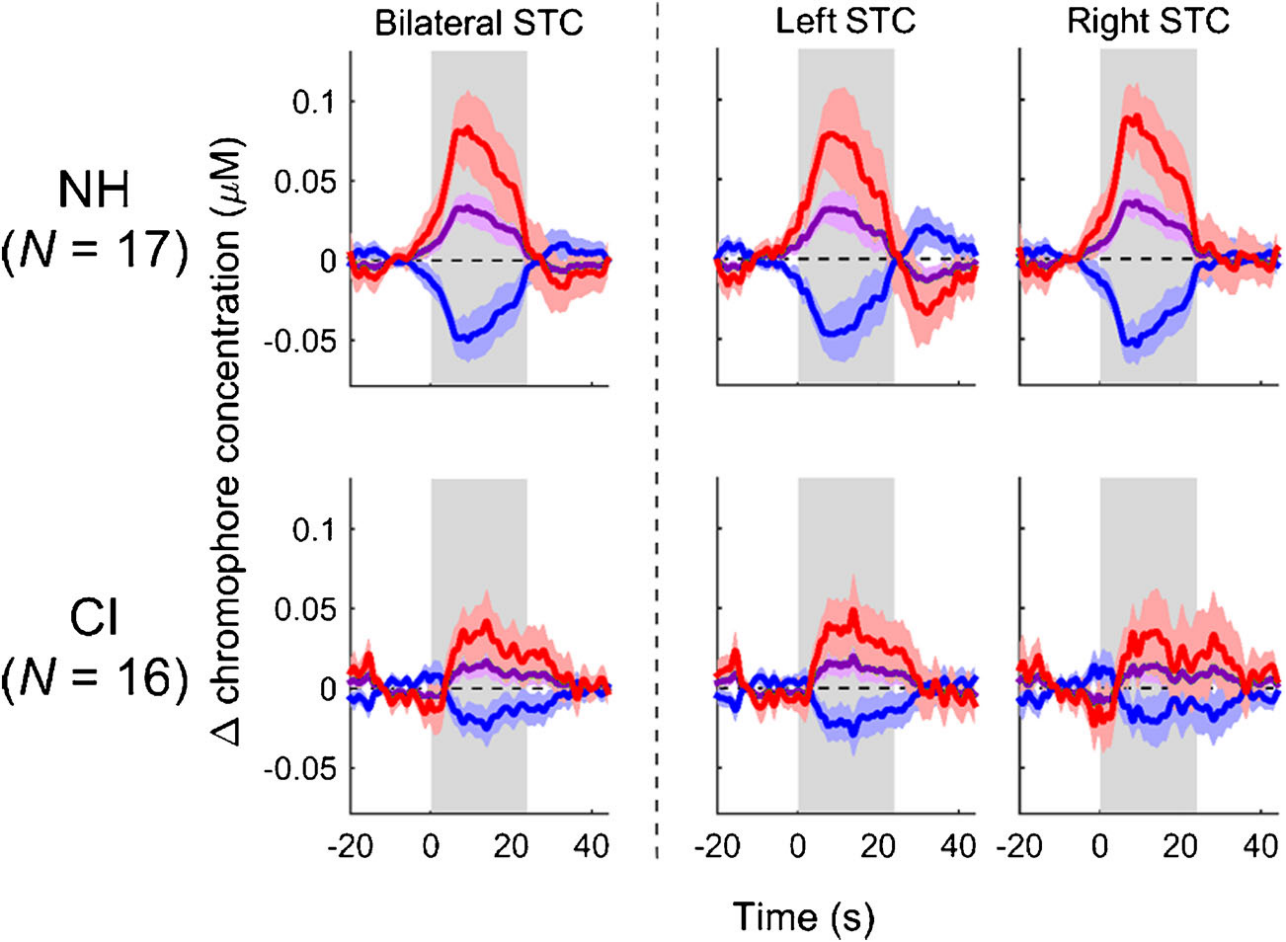
Group-averaged time courses of cross-modal activation to visual speech. Changes in HbO (red) and HbR (blue) concentration, as well as HbT levels (purple), during the presentation of visual speech (stimulation period indicated by shaded grey bar) shown for normal-hearing controls (labelled NH) and CI users before implantation (labelled CI), panelled by ROI. Coloured shading indicates ± 1 standard error across participants

We then further examined cross-modal activation of bilateral STC by visual speech to better understand what the activity may represent. Figure 3 displays pre-operative activation patterns across the optode arrays using contrast image data. As can be seen here, cortical activations to visual speech (compared to rest) were largely non-significant across both CI and NH participants. Plotting the group-averaged time courses in the bilateral STC ROI revealed that plausible haemodynamic responses to visual speech were measured both in deaf individuals prior to implantation and NH control subjects (Fig. 4). Figure 4 shows evidence of substantial between-subject variability in the amplitude of cortical activation to visual speech in both groups. These findings of non-significant and variable response amplitudes to visual speech are largely consistent with fMRI evidence, suggesting that these cortical response features may reflect individual variability in the speechreading networks of both NH (Hall et al. 2005) and profoundly deaf adults (Macsweeney et al. 2001).

**Fig. 5:**
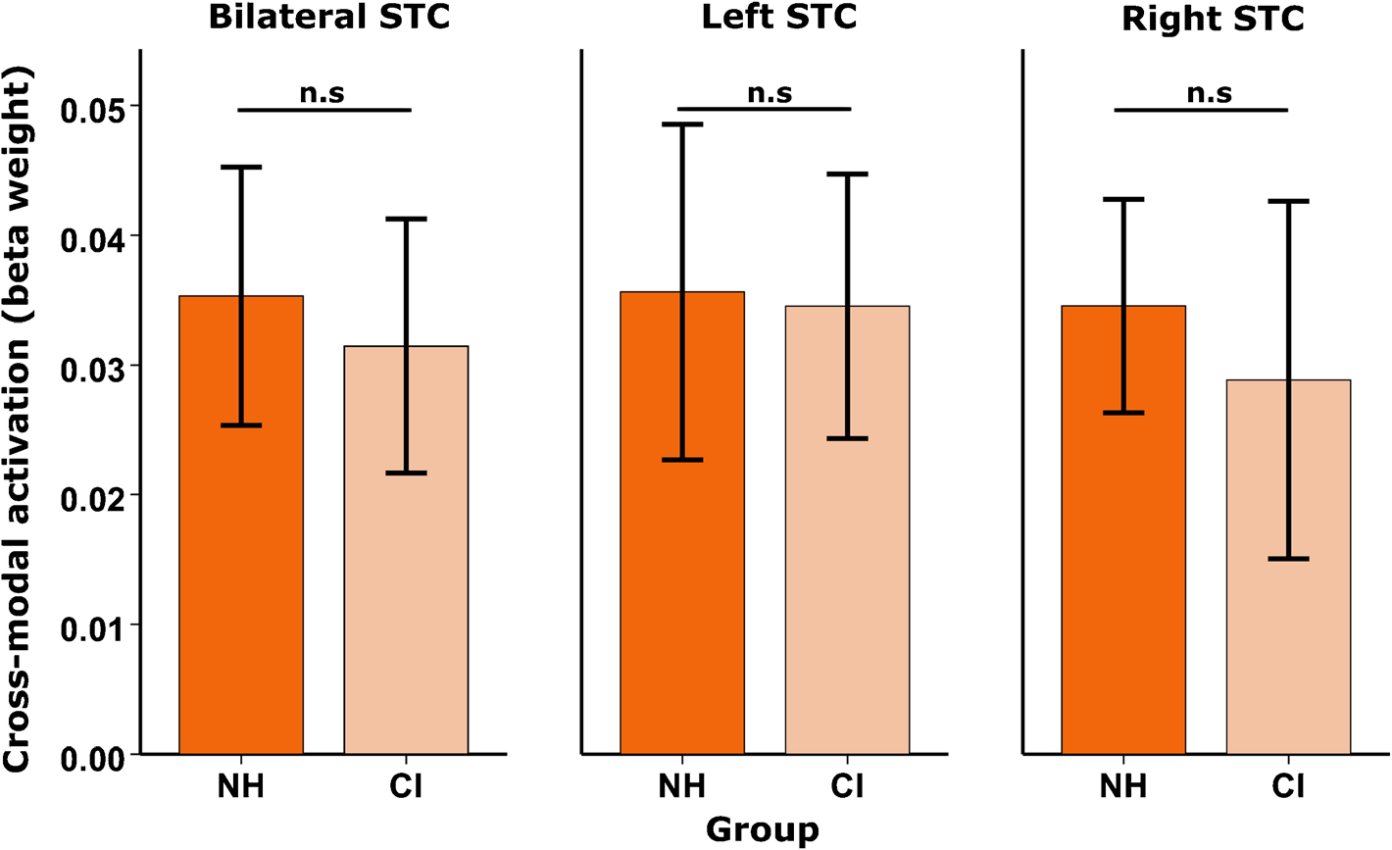
Mean amplitude of cross-modal activation to visual speech. Bar graph showing mean amplitude of cross-modal activation to visual speech (beta weight) for normal-hearing controls (NH, *n* = 17) and CI users before implantation (CI, *n* = 16), panelled by ROI. Error bars represent ± 1 standard error. n.s. non-significant

To examine whether cortical activations to visual speech differed between deaf individuals and control subjects, we conducted an independent samples *t* test on the mean amplitude of bilateral STC response. This analysis showed no evidence of a significant group difference in amplitude of bilateral STC activation (*t*_(31)_ = .28, *p* = .79, 2-tailed; Fig. 5). Inspection of the left and right hemisphere separately also revealed no evidence of a significant difference in cortical activation between the two groups (left *t*_(31)_ = .07, *p* = .94; right *t*_(31)_ = .36, *p* = .72, both 2-tailed; Fig. 5). Therefore, the level of cortical activation to visual speech within auditory brain regions does not seem to be enhanced in deaf subjects, compared with NH individuals.

**Fig. 6:**
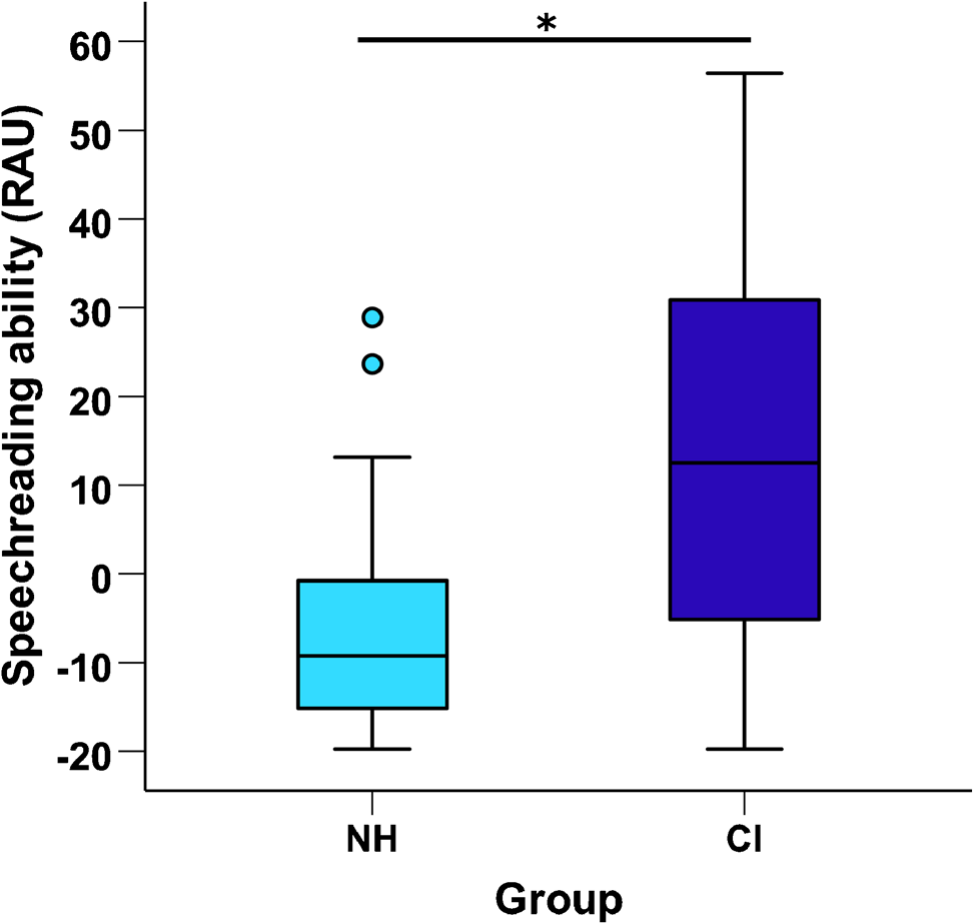
Speechreading ability in control subjects and CI users before implantation. Box plot displaying speechreading ability (words correctly identified, RAU) for normal-hearing controls (NH, *n* = 17) and CI users (CI, *n* = 17) before implantation. *p = .01, 2-tailed

**Fig. 7:**
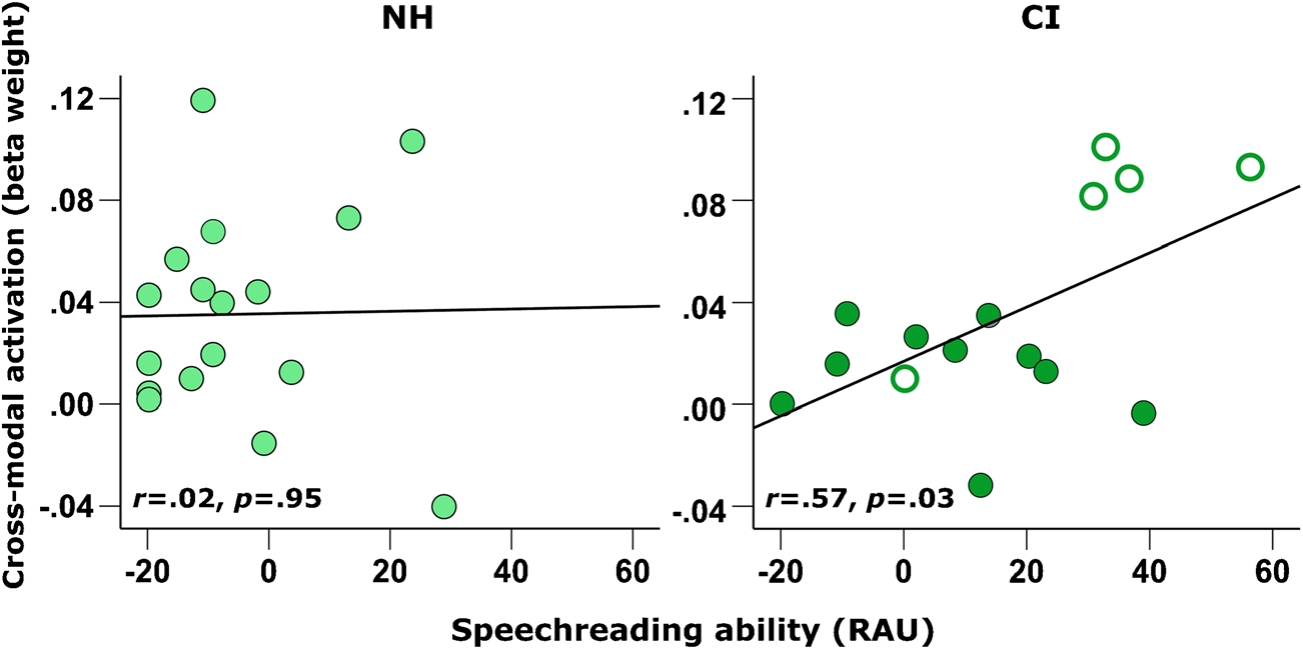
Pre-implant STC activation to visual speech and speechreading ability. Scatterplot of pre-implant bilateral STC activation to visual speech and speechreading ability with regression lines shown, panelled by group NH (*n* = 17) and CI (*n* = 15). Filled markers represent data obtained from post-lingually deaf CI users, and open markers represent data obtained from pre- and peri-lingually deaf CI users

**Fig. 8:**
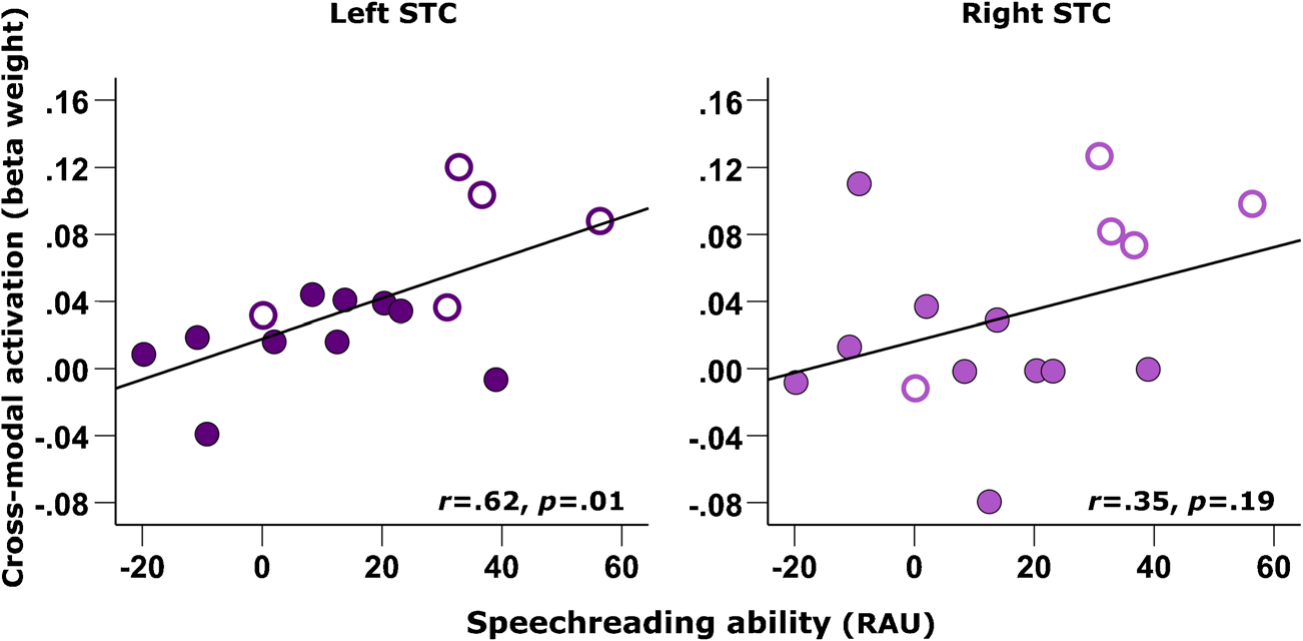
Correlation between left and right STC activation and speechreading ability in CI users. Scatterplot of pre-implant STC activation to visual speech and speechreading ability in CI users (*n* = 15) with regression line shown, panelled by ROI. Filled markers represent data obtained from post-lingually deaf CI users, and open markers represent data obtained from pre- and peri-lingually deaf CI users

Whilst no group-difference in STC activation to visual speech was observed, a Mann-Whitney *U* test indicated that a significant group difference in speechreading ability did exist (*U* = 73.5, *z* = − 2.45, *p* = .01, 2-tailed; Fig. 6), with deaf individuals prior to implantation displaying greater speechreading abilities (median = 12.5 RAUs, *n* = 17) compared to NH controls (median = − 9.2 RAUs, *n* = 17). Furthermore, correlational analysis revealed that pre-implant speechreading ability was positively associated with pre-implant bSTC activation to visual speech in the CI group (*r* = .57, *p =* .026, 2-tailed, *n* = 15; Fig. 7). Further exploration of this relationship showed that this positive association existed in the left hemisphere (*r* = .62, *p =* .013, 2-tailed, *n* = 15; Fig. 8) but not in the right hemisphere (*r* = .35, *p =* .19, 2-tailed, *n* = 15; Fig. 8), in line with the suggestion that the left STC maintains its linguistic function during deafness regardless of the sensory input modality (Cardin et al. 2013). Conversely, there was no evidence of such a relationship between bilateral STC activation to visual speech and speechreading ability in the NH control group (*r* = .02, *p* = .95, 2-tailed, *n* = 17; Fig. 7). Therefore, greater STC activation to lip-reading may reflect a cortical adaptation in deaf individuals that provides a functional benefit by supporting better speechreading abilities and which is predominately lateralised to the left hemisphere.

**Fig. 9:**
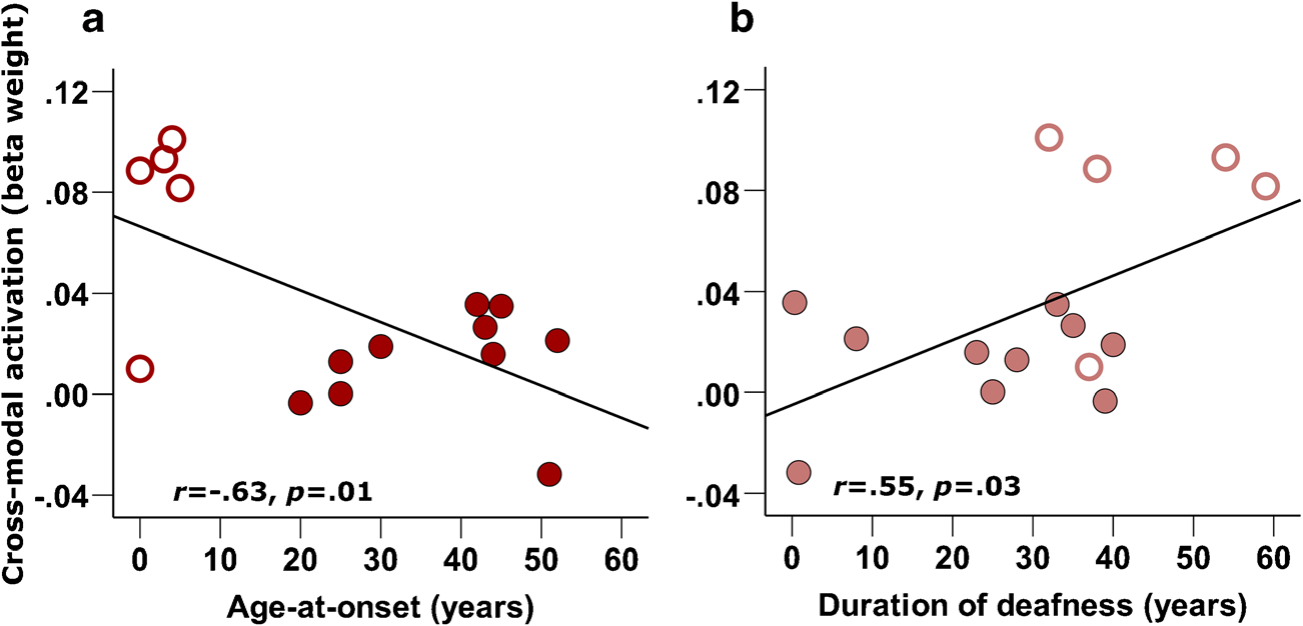
Correlations between cross-modal activation and clinical history of deafness. Scatterplot of pre-implant bilateral STC activation to visual speech with **a** age-at-onset of bilateral hearing loss and **b** duration of bilateral hearing loss, with regression lines shown (*n* = 15). Filled markers represent data obtained from post-lingually deaf CI users, and open markers represent data obtained from pre- and peri-lingually deaf CI users

Further to this, bSTC activation to visual speech was seen to be negatively correlated with the age-at-onset of bilateral hearing loss (*r* = − .63, *p =* .013, 2-tailed, *n* = 15; Fig. 9a) and was positively correlated with the duration of bilateral hearing loss (*r* = .55, *p =* .034, 2-tailed, *n* = 15; Fig. 9b). That is, a greater amplitude of bSTC activation to visual speech was associated with an earlier onset and a longer duration of auditory deprivation. Therefore, the level of pre-implant cortical activation to visual speech within STC is associated with the patients’ history of auditory deprivation.

## Discussion

A clinically viable objective tool that can help to more accurately predict outcomes following cochlear implantation is needed for use with adult CI recipients in order to better counsel their expectations and to help make more informed treatment decisions. Here we report neuroimaging and behavioural evidence from deaf adult CI candidates, indicating that fNIRS measurements of cross-modal activation to visual speech within auditory brain regions obtained pre-operatively can provide additional prognostic information about future CI outcome. Specifically, stronger pre-operative cross-modal activation of auditory brain regions by visual speech was predictive of poorer auditory speech understanding after implantation. However, this relationship appeared to be driven by group differences between pre- and post-lingually deaf individuals. Whilst the results suggest that, in principle, measures of cortical activation acquired before implantation could aid in the more accurate prognosis of CI outcome, if such cortical recordings are to be usefully applied in clinical practice, the sensitivity and specificity of the measure to predict good and poor CI outcome in individual candidates must first be established in a larger sample.

There is significant heterogeneity within adult CI-using clinical populations (e.g. Blamey et al. 2013; Lazard et al. 2010, 2012a), and so a heterogeneous group of CI candidates were recruited to this study in order to best represent a typical clinical sample. Participants were also tested in their best-aided condition as this enabled measurement of real-world, functional outcomes with a CI. Whilst these differences in aiding amongst participants (see Table 1) could influence analysis of bilateral auditory activations, the current study focusses on bilateral cortical activation to silent visual speech (with no auditory stimuli present), and so this potential confound did not pose concern. Subsequently, the current sample consisted of serial patients listed for implant surgery from the Nottingham Auditory Implant Programme that included pre- and post-lingually deaf adult CI recipients, regardless of their duration of deafness, hearing aid history and deafness aetiology. Analysis of this heterogeneous group indicated that stronger pre-operative cross-modal activation of auditory brain regions by visual speech was predictive of poorer auditory speech understanding after implantation. However, further investigation of the subgroup of post-lingually deaf individuals only showed that this relationship may be driven by group differences between pre- and post-lingually deaf individuals.

Indeed, it has been established that pre- and post-lingually deaf individuals may show different patterns of cortical reorganisation and levels of speech understanding with a CI. For instance, we know from existing studies that pre-lingually deaf subjects show greater cross-modal reorganisation in bilateral temporal lobes (Lee et al. 2001; Finney et al. 2001; Kral and Sharma 2012), which is linked to poor CI outcome (Buckley and Tobey 2011). Furthermore, it is well established that a number of variables including the age-at-onset and duration of deafness can affect speech outcomes in adults with a CI (Blamey et al. 2013; Lazard et al. 2010, 2012a; Summerfield and Marshall 1995). However, together, such known variables only account for a small proportion of variance in speech outcomes with a CI, and up to 80 % of the variance remains unaccounted for in post-lingually deaf individuals (Lazard et al. 2012a).

As the predictive relationship observed here across the whole group appeared to be largely driven by such interrelated confounding factors, these were subsequently examined. Specifically, our analysis examined whether bilateral STC activation to visual speech before implantation was able to offer any predictive value above that already provided by influential clinical characteristics of the listener (see Tables 4, 5, and 6), including the age at onset of deafness, duration of deafness and speechreading ability. Both negative and positive associations have been reported between speechreading ability and CI outcome (Hay-McCutcheon et al. 2005; Gantz et al. 1993, respectively). Here, we observed a negative correlation between pre-implant speechreading proficiency and post-implant auditory performance (*r* = − .40, *p* = .14, 2-tailed). Although this correlation did not reach statistical significance, the coefficient is consistent with a moderate correlation and thus was likely lacking power due to the small sample (*n* = 15). Whilst assessing speechreading ability would offer a simpler way of providing prognostic information compared to neuroimaging, here we show that fNIRS was able to provide unique predictive value (40 %) over that explained by pre-operative speechreading ability. Furthermore, a national study conducted in a large heterogeneous population has previously reported no evidence of a relationship between pre-implant speechreading ability and CI outcome (*r* = .16; Summerfield and Marshall 1995). Therefore, the value of speechreading proficiency as a pre-operative measure of post-operative outcome remains uncertain.

Amongst the clinical covariates examined here, the age-at-onset of bilateral HL was the only non-cortical factor that was able to significantly predict future CI outcome and was seen to correlate most highly with amplitude of STC activation to visual speech. Importantly, the current findings indicated that pre-operative activation to visual speech measured using fNIRS was able to provide significantly more and unique predictive value above the age-at-onset of bilateral HL, duration of deafness and pre-implant speechreading ability. Thus, pre-implant imaging using fNIRS could offer objective, supplementary prognostic information that could help to improve upon the accuracy and reliability of current clinical predictions of CI outcome. However, due to sample-size limitations, it was beyond the scope of the current study to establish whether the fNIRS cortical measure could offer further explanatory power above all of these clinical factors combined. Further studies examining larger groups of pre-lingually deaf adults and post-lingually deaf adults separately would help to elucidate any potential links between the extent of cross-modal plasticity in auditory areas and CI outcomes.

In order to gain mechanistic insight into this unique predictive ability of the pre-operative fNIRS measurements, we examined what pre-implant cross-modal activation to visual speech may have reflected. Existing reports show that adults with early-onset (Auer et al. 2007; Bernstein et al. 2000; Ellis et al. 2001) and late-onset deafness (Rouger et al. 2007) display greater speechreading abilities compared to NH listeners. Likewise, here we show that deaf individuals were more proficient at speechreading compared to NH control subjects, providing an adaptive strategy to aid spoken communication during deafness. Neuroimaging studies have investigated whether differences in cortical activations to visual speech underlie this behavioural adaptation to deafness. Whilst greater levels of bilateral STC activation to visual speech have been demonstrated in congenitally (Capek et al. 2008) and post-lingually deafened individuals compared to NH control subjects (Lee et al. 2007), conversely, this group difference has also been demonstrated in the opposite direction (MacSweeney et al. 2002). Furthermore, evidence tells us that each hemisphere has its own specificity, in particular regarding speech processing (Cardin et al. 2013; Hall et al. 2005; Lazard et al. 2012b; Zatorre and Belin 2001), and so as well as examining bilateral activation, we also examined each hemisphere separately.

Here we found no evidence of a group difference in either direction in the level of bilateral STC activation to visual speech. However, correlational analysis did reveal that greater cortical activation to visual speech, in the left but not the right hemisphere, was related to better speechreading ability in deaf individuals, whereas no such relationship existed in NH control subjects. Thus, greater recruitment of superior temporal brain regions by visual speech in the absence of reliable auditory input appears to provide a functional benefit for deaf individuals by supporting better speechreading abilities. Furthermore, correlational analysis indicated that greater cortical activation to visual speech was associated with a longer duration and earlier age-at-onset of auditory deprivation, suggesting that this cortical adaptation may develop as a function of the patient’s clinical history of deafness. Our findings corroborate previous fMRI evidence that greater responsivity to visual speech within the left posterior superior temporal brain region is functionally related to greater speechreading ability in profoundly deaf individuals, whereas greater responsivity to visual speech within the right posterior superior temporal brain regions appears to offer no such communicative advantage (Capek et al. 2008; Capek et al. 2010; Lee et al. 2007). Our findings support the notion that, in the absence of auditory input, the left STC may still retain its linguistic function regardless of the sensory input modality (Cardin et al. 2013).

Whilst greater pre-implant STC activation to visual speech appears functionally advantageous during deafness, conversely, it has been speculated that the processing of non-linguistic visual stimuli (Buckley and Tobey 2011; Doucet et al. 2006; Lee et al. 2001; Sandmann et al. 2012) and visual speech (Rouger et al. 2012; Strelnikov et al. 2013) within temporal brain regions of CI users negatively influences CI outcome through a deleterious effect on the ability of the auditory brain regions to respond to auditory stimulation. However, here, the data provide no evidence that responsiveness of bilateral STC to visual speech before implantation was inversely related to the responsiveness of bilateral STC to auditory speech after implantation. Thus, the current findings provide no evidence to suggest that greater recruitment of auditory brain regions for processing visual speech during deafness limits the future capacity of these brain regions to respond to auditory speech when later stimulated with a cochlear implant. Whilst the current study focusses on understanding the link between brain organisation before implantation and future CI outcome, the findings are somewhat complementary to recent longitudinal evidence of changes in brain activation observed from before to after implantation, which shows that the auditory and visual modality do not compete against each other but rather work cooperatively following cochlear implantation (Anderson et al. 2017b). Furthermore, that responsiveness of auditory brain regions to cochlear implant stimulation is not substantially affected by cross-modal reorganisation has been demonstrated previously in a cortical area involved in cross-modal function in congenitally deaf animals (Land et al. 2016). It should be noted in the current study that fNIRS provides only an indirect measure of cortical activation and the trade-off between visual and auditory activation (or rather, its absence). It is therefore difficult to make firm conclusions about the cortical mechanisms using the fNIRS technique alone. However, the aforementioned supporting evidence from animal models, including in vivo neuron recordings, does provide complementary evidence to support the current argumentation and findings in humans presented here.

Although the current study aimed to quantify CI outcome as the level of auditory speech perception ability in quiet following implantation, the results indicated that some participants performed at or near to ceiling. Therefore, for some individuals, it was not possible to accurately or fully estimate their level of auditory performance with a CI due to the constraints of speech perception testing in quiet conditions and use of a percent correct measurement scale. Future research should consider employing a more sensitive test, such as speech perception testing in noise. However, it is important to note potential problems associated with using such methods with CI users, including participant listening discomfort, de-motivation and/or emotional distress. Use of more ecologically valid tests would improve the validity and generalisability of future findings.

## Conclusions

Significant heterogeneity exists within adult CI-using clinical populations. Although a number of clinical characteristics are known to influence CI outcome, a large proportion of variance still remains unexplained and may be accounted for by brain reorganisation during the period of deafness. This study investigated whether pre-operative imaging of auditory brain regions using fNIRS could help to explain a proportion of the remaining variability and improve upon the accuracy and reliability of prognostic information that is currently available to CI candidates and their clinical team. The current findings in a heterogeneous group of pre- and post-lingually deaf CI users provide evidence of a predictive relationship between activation of temporal brain regions by visual speech before implantation and future auditory speech understanding with a CI following 6 months of use. This negative relationship appeared to be driven by the subgroup of pre-lingually deaf individuals. Whilst it was apparent that this relationship was influenced by other interrelated confounding factors, including the age-at-onset of deafness, duration of deafness and speechreading ability, subsequent analyses indicated that pre-operative cortical imaging was able to provide significant predictive value above that provided by these influential clinical characteristics. Thus, the use of fNIRS as an objective measure prior to cochlear implantation may enable us to deliver more accurate prognostic information to adult CI candidates.

Cortical activation of left auditory brain regions by visual speech prior to implantation was positively associated with speechreading ability in deaf, but not hearing, individuals. This demonstrates that, whilst the sensory modality of cortical regions may change during deafness (i.e. from audition to vision), these regions maintain their function (i.e. specialisation for language processing), supporting enhanced speechreading proficiency during periods of deafness. Activation of auditory brain regions by visual speech prior to implantation was not related to future level of cortical activation evoked by auditory speech stimulation with a cochlear implant but was negatively related to the age-at-onset of deafness and positively related to the duration of deafness. These findings indicate that activation of auditory brain regions by visual speech prior to implantation (i) may help to maintain the linguistic specialisation of left temporal lobe regions during periods of deafness, (ii) does not negatively impact on the ability of these brain regions to respond to future auditory stimulation with a CI and (iii) is influenced by the CI user’s clinical history of deafness.
